# Pediatric MASLD in the precision medicine era: advances in diagnosis, prevention, and treatment

**DOI:** 10.3389/fmed.2026.1927710

**Published:** 2026-10-15

**Authors:** Anastasia Ntikoudi, Anastasia Papachristou, Eugenia Vlachou

**Affiliations:** 1 Department of Nursing, University of West Attica, Athens, Greece; 2 Department of History, Politics and International Studies, Neapolis University Pafos, Pafos, Cyprus

**Keywords:** adolescents, cardiometabolic risk, children, insulin resistance, metabolic dysfunction-associated steatotic liver disease, non-invasive diagnosis, obesity, pediatric MASLD

## Abstract

Metabolic dysfunction-associated steatotic liver disease (MASLD) has emerged as one of the most clinically relevant chronic liver diseases in children and adolescents, reflecting the convergence of pediatric obesity, insulin resistance, dyslipidemia, altered glucose metabolism, and broader cardiometabolic risk. The replacement of non-alcoholic fatty liver disease (NAFLD) with MASLD represents a substantive conceptual shift, moving the field away from an exclusion-based and potentially stigmatizing definition toward a pathophysiologically grounded framework centered on metabolic dysfunction. This transition is particularly important in pediatric hepatology, where steatotic liver disease is rarely defined by alcohol exposure and is more commonly embedded within complex developmental, familial, behavioral, genetic, and socioeconomic determinants of metabolic health. Although pediatric MASLD is often discussed as part of the obesity epidemic, its clinical heterogeneity indicates that body mass index alone is insufficient to capture disease risk, severity, or prognosis. Recent evidence suggests that children with MASLD may be stratified into distinct metabolic and inflammatory phenotypes, supporting a move from uniform management toward risk-adapted and phenotype-informed care. This review synthesizes current advances in the diagnosis, prevention, and treatment of pediatric MASLD, with emphasis on precision medicine as a framework for integrating clinical, biochemical, imaging, genetic, lifestyle, and social determinants into individualized care pathways. Current management remains anchored in lifestyle intervention and multidisciplinary cardiometabolic risk reduction, while important gaps persist in pediatric-specific non-invasive diagnostics, fibrosis risk prediction, longitudinal monitoring, and MASLD-targeted pharmacotherapy. A precision medicine approach may improve early identification of high-risk children, guide intensity of follow-up, inform therapeutic prioritization, and ultimately reduce liver-related and cardiometabolic complications across the life course.

## 1 Introduction

Metabolic dysfunction-associated steatotic liver disease (MASLD) has become an increasingly important pediatric condition, representing both a hepatic manifestation of metabolic dysfunction and an early-life marker of systemic cardiometabolic vulnerability (1). Its epidemiological burden varies according to population characteristics, diagnostic modality, and disease definition. Lee et al. (2) estimated a worldwide pooled prevalence of pediatric NAFLD of 13% in the general population and 47% among children with obesity, while Jia et al. (3) reported a pooled prevalence of 41.2% for metabolic dysfunction-associated fatty liver disease among children and adolescents with overweight or obesity. Despite methodological heterogeneity, these estimates demonstrate that pediatric steatotic liver disease is a clinically significant component of the childhood obesity and metabolic dysfunction epidemic and requires multidisciplinary attention from clinical and public health professionals (4).

The 2023 multisociety Delphi consensus replaced non-alcoholic fatty liver disease (NAFLD) with MASLD to move away from an exclusion-based definition and better reflect the metabolic pathogenesis of hepatic steatosis (5). This terminology is particularly relevant in pediatrics, where disease is primarily associated with obesity, insulin resistance, dyslipidemia, altered glucose metabolism, pubertal metabolic changes, lifestyle exposures, family history, and social determinants rather than alcohol consumption (6, 7). Nevertheless, the transition creates challenges for evidence synthesis because much of the pediatric literature was generated using NAFLD or metabolic dysfunction-associated fatty liver disease definitions. Studies must therefore specify the terminology and diagnostic criteria applied and distinguish clearly among steatosis, steatohepatitis, fibrosis, and extrahepatic metabolic outcomes.

Pediatric MASLD should not be regarded simply as adult MASLD occurring at an earlier age. Growth, puberty, hormonal regulation, body-fat distribution, family dependence, school environments, and evolving lifestyle behaviors influence disease development and clinical expression. Histological patterns may also differ from those observed in adults, including differences in the distribution of inflammation and fibrosis (1, 7). Consequently, the direct application of adult screening algorithms, fibrosis scores, therapeutic evidence, or trial endpoints to children may result in misclassification or inappropriate management (8). Pediatric MASLD therefore requires developmentally appropriate diagnostic thresholds, outcome measures, and care pathways.

The clinical significance of pediatric MASLD extends beyond hepatic fat accumulation. Affected children frequently present with obesity, insulin resistance, dyslipidemia, impaired glucose metabolism, hypertension, obstructive sleep apnea, and other obesity-related comorbidities (9). Hepatic steatosis may therefore indicate a broader adverse metabolic phenotype rather than an isolated liver abnormality (9). Clinical assessment should integrate liver-related findings with cardiometabolic risk, family history, lifestyle exposures, psychosocial context, and access to preventive resources (10). This systemic perspective places pediatric MASLD at the intersection of hepatology, endocrinology, nutrition, behavioral medicine, and public health.

Diagnosis and treatment remain constrained by an incomplete pediatric evidence base. Alanine aminotransferase is accessible but lacks sufficient sensitivity and specificity to identify steatosis, steatohepatitis, or fibrosis when used alone (10). Conventional ultrasound has limited sensitivity for mild steatosis and cannot reliably quantify liver fat or stage fibrosis, whereas MRI-proton density fat fraction and elastography-based modalities provide more refined assessment but remain limited by cost, availability, standardization, and insufficiently validated pediatric thresholds (11). These limitations support multimodal, risk-adapted assessment rather than reliance on a single test. Lifestyle intervention remains the foundation of treatment, but its effectiveness depends on family circumstances, school and community environments, socioeconomic resources, mental health, stigma, health literacy, and the feasibility of sustained dietary and physical-activity changes (12). Dietary and exercise interventions show potential benefit, although studies remain heterogeneous in design, duration, adherence support, and liver-specific endpoints (13). Pharmacological options are also evolving, particularly through obesity-directed and incretin-based therapies, but improvements in body weight or metabolic risk should not be interpreted as established MASLD-specific disease modification in children (14).

The marked heterogeneity of pediatric MASLD provides the central rationale for precision medicine. Children with similar body mass index may differ substantially in visceral adiposity, insulin sensitivity, lipid metabolism, genetic susceptibility, inflammatory activity, fibrosis risk, gut–liver axis characteristics, behavioral exposures, and social vulnerability (15, 16). Huneault et al. (17) used unsupervised clustering of clinical and metabolomic data from 514 children with biopsy-confirmed MASLD and identified early-mild, cardiometabolic, and inflammatory-fibrotic metabotypes. These findings support the view that pediatric MASLD is not a uniform disease and that monitoring and treatment intensity may ultimately need to be adapted to clinically meaningful phenotypes. Precision medicine in this context does not necessarily require immediate routine use of omics technologies. Rather, it begins with the structured integration of clinical phenotype, metabolic risk, biomarkers, imaging, family context, and social determinants to support individualized and equitable care (10).

This review aimed to synthesize recent advances in the diagnosis, prevention, and treatment of pediatric MASLD in the precision medicine era. The review examines the implications of the new nomenclature, the evolving epidemiology of pediatric steatotic liver disease, the cardiometabolic architecture of risk, current and emerging diagnostic tools, family-centered and public health prevention strategies, lifestyle and nutritional interventions, obesity-directed pharmacotherapy, and the potential role of genetics, microbiome science, digital health, and data-driven risk stratification. By integrating these areas, the review argues that pediatric MASLD should be approached as a heterogeneous, developmentally embedded, cardiometabolic liver disease requiring earlier detection, individualized monitoring, multidisciplinary intervention, and equity-sensitive implementation.

### 1.1 Literature search and evidence selection

This narrative review was informed by a structured literature search conducted in PubMed, Scopus, CINAHL, the Cochrane Library, and MEDLINE. The searches were performed between May 2026 and June 30, 2026. Search terms related to pediatric populations and steatotic liver disease included “child,” “children,” “adolescent,” “pediatric,” “metabolic dysfunction-associated steatotic liver disease,” “MASLD,” “metabolic dysfunction-associated fatty liver disease,” “MAFLD,” “non-alcoholic fatty liver disease,” “NAFLD,” “steatohepatitis,” and “NASH.” These terms were combined with topic-specific terms relating to epidemiology, cardiometabolic risk, pathophysiology, diagnosis, non-invasive assessment, prevention, lifestyle intervention, pharmacotherapy, precision medicine, genetics, microbiome, digital health, and machine learning.

The reference lists of relevant clinical guidelines, practice statements, systematic reviews, meta-analyses, and major clinical studies were also examined to identify additional publications. Priority was given to pediatric clinical guidelines and consensus statements, systematic reviews and meta-analyses, randomized controlled trials, cohort studies, diagnostic-validation studies, and recent studies directly addressing pediatric MASLD phenotypes or precision-medicine approaches. Adult studies were included selectively when they provided essential regulatory, therapeutic, mechanistic, or long-term safety context for which pediatric evidence was unavailable. Studies were considered for detailed narrative synthesis when they included children or adolescents with NAFLD, MAFLD, or MASLD, or provided pediatric-specific evidence relevant to at least one of the predefined domains of the review. Evidence exclusively derived from adults was not used to support pediatric clinical recommendations and was incorporated only when pediatric data were unavailable for regulatory, mechanistic, therapeutic, or long-term safety considerations. Study selection was guided by direct relevance to pediatric MASLD, methodological strength, recency, and clinical applicability.

Evidence was synthesized narratively according to study design, population, diagnostic definition, sample size, comparator, measured outcomes, duration of follow-up, directness to pediatric MASLD, and clinical applicability. Particular attention was given to differences among studies using NAFLD, MAFLD, or MASLD terminology. This review was designed as a structured narrative review rather than a systematic review; therefore, no formal meta-analysis, standardized risk-of-bias instrument, or certainty-of-evidence grading framework was applied.

To conceptualize the integration of diagnosis, prevention, treatment, and follow-up, Figure 1 presents a precision-medicine framework for pediatric MASLD.

**Figure 1:**
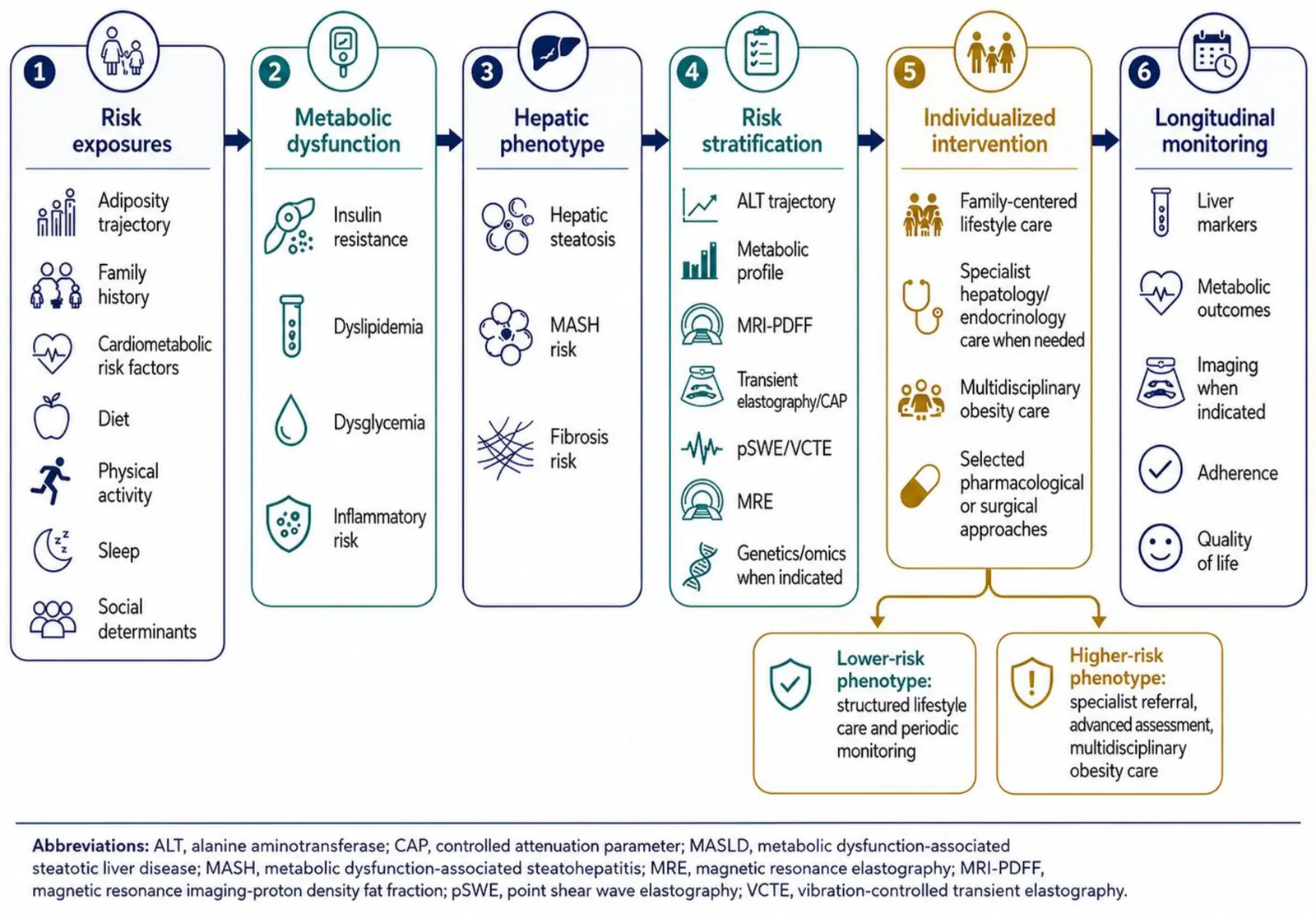
Precision-medicine framework for pediatric MASLD.

Precision medicine in pediatric MASLD integrates biological, metabolic, behavioral, imaging, and social determinants to support risk-adapted care. The framework progresses from early risk identification and metabolic characterization to hepatic phenotyping, individualized intervention, and longitudinal monitoring. Lower-risk children may receive structured family-centered lifestyle care and periodic monitoring, whereas higher-risk children may require specialist assessment, advanced imaging, multidisciplinary obesity care, and selected pharmacological or surgical interventions (7, 17).

## 2 From NAFLD to MASLD: conceptual and clinical implications for pediatrics

The replacement of NAFLD with MASLD reflects a shift from exclusion-based terminology toward pathophysiological classification. NAFLD defined hepatic steatosis primarily by the absence of substantial alcohol intake and other secondary causes (18). As the metabolic basis of the disease became clearer, this terminology was increasingly considered inadequate. The multisociety Delphi consensus led by Rinella et al. therefore selected MASLD to describe steatotic liver disease occurring in the presence of cardiometabolic risk factors and to address potentially stigmatizing aspects of the NAFLD/NASH terminology (5). In pediatrics, this change aligns more closely with the clinical reality that hepatic steatosis commonly occurs alongside obesity, insulin resistance, and related metabolic abnormalities (19).

The implications are particularly important because pediatric MASLD develops within the context of growth, puberty, neurodevelopment, family dependence, school routines, and evolving lifestyle behaviors (6). The pediatric multisociety statement emphasized that steatotic liver disease in children has age-specific characteristics and should not be managed solely according to adult paradigms (6). Developmental differences affect liver-enzyme thresholds, body composition, pubertal insulin resistance, imaging interpretation, treatment participation, and adherence to long-term care (20).

The terminology transition also complicates evidence synthesis. Studies conducted under NAFLD, NASH, MAFLD, or MASLD definitions may include overlapping but not identical populations, particularly when cardiometabolic criteria, secondary causes, and diagnostic methods differ. Pediatric studies should therefore define the terminology and criteria applied, specify how steatosis was identified, and distinguish liver-related outcomes, including steatosis, steatohepatitis, and fibrosis, from extrahepatic outcomes such as insulin resistance, dyslipidemia, and type 2 diabetes risk (5, 7). Consistent reporting is essential for reliable comparison, guideline development, clinical translation, and pediatric risk prediction.

The MASLD terminology may also support clearer and less stigmatizing communication with families. Removing the terms “non-alcoholic” and “fatty” may reduce confusion and judgment, although “metabolic dysfunction” should be explained as a potentially modifiable clinical condition rather than a fixed biological defect (6). Clinicians should use non-blaming, family-centered language when discussing liver health, nutrition, physical activity, sleep, weight trajectory, insulin resistance, and long-term cardiometabolic risk (6, 7).

## 3 Epidemiology and cardiometabolic risk architecture

The epidemiology of pediatric MASLD is difficult to summarize with a single prevalence figure because estimates vary by diagnostic test, population setting, adiposity status, age, sex, geography, and terminology. Nonetheless, recent meta-analytic evidence demonstrates a substantial burden (2, 3). This burden should be interpreted cautiously because NAFLD, MAFLD, and MASLD definitions are not identical, and because studies used different diagnostic modalities, including liver enzymes, ultrasound, elastography, MRI-based methods, and biopsy-enriched cohorts (5). However, the consistency of high prevalence among children with excess adiposity makes clear that pediatric steatotic liver disease is a major clinical and public health problem (9). Concrete subgroup estimates further illustrate this epidemiological heterogeneity. Lee et al. (2) reported a pooled NAFLD prevalence of 15% in males and 10% in females in the general pediatric population, increasing to 54% and 39%, respectively, among children with obesity. Similarly, Jia et al. (3) found substantial geographical variation among children and adolescents with overweight or obesity, with MAFLD prevalence ranging from 43.6% in North America to 31.1% in Africa. These differences highlight the influence of sex, adiposity, geographic setting, and diagnostic criteria on reported prevalence estimates.

Obesity is the most visible population-level risk factor for pediatric MASLD, but it should not be treated as a complete explanation of disease risk. Body mass index is an accessible epidemiological measure, yet it does not fully capture visceral adiposity, ectopic fat distribution, insulin resistance, lipid abnormalities, inflammatory activity, genetic susceptibility, or fibrosis risk (10). This limitation is particularly important in pediatrics, where growth and pubertal development affect body composition and metabolic interpretation. Consensus and practice statements increasingly emphasize that children with suspected MASLD require assessment of the broader cardiometabolic phenotype rather than classification based only on weight category (7, 21). A precision medicine framework therefore begins with the recognition that BMI identifies risk at the population level but is insufficient for individualized prognosis.

Insulin resistance occupies a central position in the pathophysiology and clinical architecture of pediatric MASLD. Impaired insulin signaling promotes adipose tissue lipolysis, increased hepatic free fatty acid flux, compensatory hyperinsulinemia, hepatic *de novo* lipogenesis, altered glucose metabolism, and inflammatory signaling, thereby linking hepatic steatosis to systemic metabolic dysfunction. In clinical terms, this means that a child with suspected MASLD should be assessed not only for liver injury but also for prediabetes, type 2 diabetes risk, dyslipidemia, hypertension, and other obesity-associated comorbidities (7, 21). The liver may therefore serve as an early indicator of metabolic disease progression, particularly in children with severe obesity, family history of type 2 diabetes, acanthosis nigricans, or worsening glycemic markers.

The burden of pediatric MASLD is also shaped by sex, developmental stage, ethnicity, geography, and socioeconomic context. Meta-analytic data suggest sex-related differences in prevalence, with higher estimates often reported in boys, while adolescence appears to be a particularly important period because pubertal insulin resistance, hormonal changes, behavioral autonomy, sleep disruption, and sedentary patterns may intensify metabolic risk (22). At the same time, structural determinants such as access to healthy food, safe physical activity, preventive health care, laboratory testing, imaging, and pediatric specialist services influence both disease risk and detection (12). These factors are not peripheral to MASLD; they are part of the causal and clinical architecture of the disease (12). Any precision medicine model that incorporates genetic or imaging data but ignores social determinants risks producing technically sophisticated but inequitable care pathways (12).

The cardiometabolic clustering observed in pediatric MASLD has implications for long-term health (17). Although not every child with steatosis will progress to advanced liver disease, the presence of MASLD during childhood may identify a subgroup with increased lifetime exposure to insulin resistance, dyslipidemia, systemic inflammation, and cardiometabolic risk. Recent pediatric guidance therefore emphasizes integrated assessment and management rather than liver-only follow-up (1). This approach is consistent with a life-course model in which pediatric MASLD is not viewed as an isolated childhood diagnosis, but as a potentially early manifestation of metabolic disease trajectories that may extend into adulthood (1). Prevention and treatment should therefore aim not only to reduce liver fat, but also to modify the broader metabolic environment in which liver injury develops.

## 4 Pathophysiological basis of pediatric MASLD

Pediatric MASLD is best understood through a multiple-hit framework in which metabolic, genetic, inflammatory, microbial, developmental, behavioral, and environmental factors interact to produce hepatic steatosis, steatohepatitis, and fibrosis in susceptible children (23). Although excess adiposity is a major driver, children with similar body mass index may differ considerably in visceral adiposity, insulin sensitivity, hepatic lipid handling, inflammatory activity, genetic susceptibility, gut microbiota composition, lifestyle exposures, and social vulnerability (23). This heterogeneity explains why pediatric MASLD cannot be regarded as a uniform consequence of obesity and supports the relevance of precision phenotyping for clinical care and research (24)(Figure 2).

**Figure 2:**
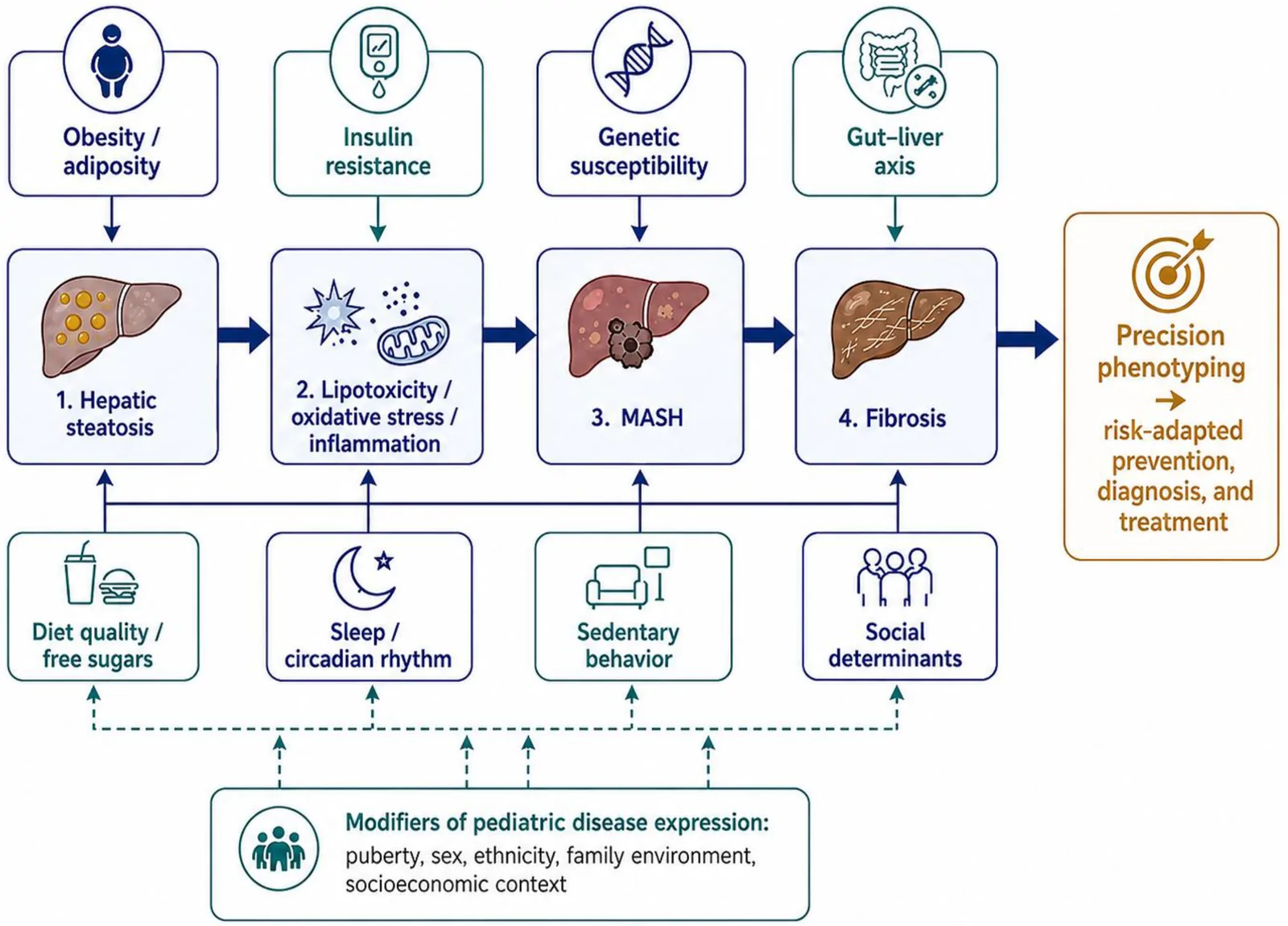
Pathophysiological and clinical heterogeneity of pediatric MASLD.

Insulin resistance is a central mechanism linking excess adiposity to hepatic steatosis. Impaired insulin action in adipose tissue increases lipolysis and free fatty acid delivery to the liver, while hepatic insulin resistance and compensatory hyperinsulinemia promote *de novo* lipogenesis and reduce metabolic flexibility (25). These changes contribute to triglyceride accumulation, altered glucose homeostasis, oxidative stress, and susceptibility to inflammatory injury (25). Insulin-resistance indices may also support non-invasive risk assessment. Song et al. (26) reported that SPISE and METS-IR performed better than some traditional measures for identifying pediatric MASLD, although their clinical utility requires further validation.

Progression from steatosis to metabolic dysfunction-associated steatohepatitis involves lipotoxicity, hepatocellular stress, and inflammatory activation (27). Although triglyceride storage may initially protect hepatocytes, excess saturated fatty acids, ceramides, diacylglycerols, cholesterol metabolites, and other toxic lipid intermediates can induce mitochondrial dysfunction, endoplasmic reticulum stress, oxidative injury, and apoptosis (27). These processes occur within the developmental context of growth, pubertal insulin resistance, hormonal change, and evolving fat distribution, which may partly explain differences between pediatric and adult disease and the limited performance of adult-derived severity models in children (7). Low-grade systemic and hepatic inflammation is increasingly recognized as an important component of pediatric MASLD and a potential target for non-invasive phenotyping. Circulating inflammatory markers, including high-sensitivity C-reactive protein, TNF-α, IL-6, and IL-18, as well as markers of hepatocyte injury and apoptosis such as cytokeratin-18, have been investigated in relation to steatosis, inflammatory activity, and disease severity (9, 28). However, many of these markers are also influenced by obesity, insulin resistance, and other inflammatory conditions. Consequently, although they may contribute to multimarker risk-stratification approaches, none currently has sufficient pediatric validation and specificity to serve as a stand-alone biomarker for routine diagnosis or longitudinal monitoring.

Fibrosis is the histological feature most closely associated with long-term liver-related risk. Persistent hepatocellular injury, oxidative stress, inflammatory signaling, and gut-derived immune activation stimulate hepatic stellate cells and extracellular matrix deposition (29). Pediatric MASLD may show more prominent portal-based inflammation or fibrosis than the classic adult zone 3 pattern (29). This distinction has implications for biopsy interpretation, validation of non-invasive fibrosis tests, trial endpoint selection, and the development of pediatric-specific risk-prediction tools (29).

The gut–liver axis and genetic susceptibility contribute further to interindividual variation. Alterations in intestinal permeability, microbial composition, short-chain fatty acids, bile acids, endogenous ethanol production, and immune signaling may promote hepatic lipid accumulation and inflammation (16). However, pediatric microbiome studies remain limited by small samples, heterogeneous sequencing methods, and confounding by obesity, diet, and metabolic comorbidities. Microbial and metabolomic signatures reported in multi-omics studies therefore remain exploratory and require external validation before clinical translation (30).

Variants affecting hepatic lipid remodeling, lipoprotein secretion, lipid-droplet biology, glucose metabolism, and inflammation, including PNPLA3, TM6SF2, GCKR, MBOAT7, HSD17B13, and MTARC1, have been associated with MASLD susceptibility or histological severity (31). In Chinese children, the PNPLA3-Ile148Met and TM6SF2-Glu167Lys variants were independently associated with MASLD, supporting the potential relevance of genotype to pediatric risk stratification (32). Quantitative evidence further supports these associations. A meta-analysis including 1,173 children with NAFLD and 1,792 controls found that the PNPLA3 rs738409 G allele was associated with approximately threefold higher odds of pediatric NAFLD (OR 3.34, 95% CI 1.52–7.33) (33). Similarly, a meta-analysis evaluating TM6SF2 rs58542926 found that the T allele was associated with increased NAFLD susceptibility in children (OR 2.87, 95% CI 1.85–4.46) (34). These findings indicate that inherited susceptibility can substantially modify hepatic risk in children exposed to otherwise similar metabolic and environmental factors. Nevertheless, routine genetic testing is not currently justified because its incremental predictive value, cost-effectiveness, ethical implications, and influence on management remain insufficiently established (23).

Early-life and developmental exposures may also influence susceptibility before abnormal liver enzymes or imaging findings become apparent. Maternal obesity, gestational diabetes, infant feeding, early weight gain, antibiotic exposure, diet, sleep, physical activity, and the household environment may affect metabolic programming, adiposity distribution, insulin sensitivity, and microbiota development (15). Precision prevention should therefore incorporate family history, perinatal exposures, growth trajectory, current metabolic status, and modifiable environmental factors (35).

Overall, pediatric MASLD represents a heterogeneous developmental-metabolic syndrome involving adiposity, insulin resistance, hepatic lipid dysregulation, lipotoxicity, inflammation, fibrosis, genetic susceptibility, gut-liver interactions, and environmental exposures (9). This complexity supports stratification according to clinically meaningful risk domains rather than reliance on body mass index or alanine aminotransferase alone (9). The metabotypes identified by Huneault et al. (17) provide proof of concept that integrated clinical and molecular phenotyping may help distinguish children with early-mild disease from those with cardiometabolic or inflammatory-fibrotic profiles, although prospective validation is required before clinical implementation.

Pediatric MASLD develops through interacting metabolic, inflammatory, genetic, microbial, developmental, behavioral, and social pathways. Excess adiposity and insulin resistance promote hepatic steatosis, while lipotoxicity, oxidative stress, mitochondrial dysfunction, inflammatory signaling, gut-derived immune activation, and genetic susceptibility may contribute to MASH and fibrosis. Disease expression is further modified by puberty, sex, ethnicity, lifestyle, family environment, and socioeconomic conditions, supporting precision phenotyping and risk-adapted care.

## 5 Advances in diagnosis of pediatric MASLD

The diagnosis of pediatric MASLD requires identification of hepatic steatosis in the presence of cardiometabolic risk, while alternative causes of steatotic liver disease should be excluded when clinically indicated (7). This differential diagnostic step is particularly important in children, in whom hepatic steatosis should not automatically be attributed to metabolic dysfunction or obesity. Alternative or coexisting causes should be considered according to age, clinical presentation, family history, medication exposure, and accompanying laboratory abnormalities. These include Wilson disease, alpha-1 antitrypsin deficiency, lysosomal acid lipase deficiency, fatty-acid oxidation and mitochondrial disorders, glycogen storage diseases, lipodystrophies and disorders of lipoprotein metabolism, as well as viral hepatitis, autoimmune liver disease, celiac disease, endocrine disorders, medication-associated steatosis, malnutrition, and parenteral nutrition (36). A broader diagnostic evaluation is particularly warranted in younger children, those without overweight or typical metabolic risk factors, and those with atypical or disproportionate biochemical or clinical findings. This process remains challenging because many children are asymptomatic, and routine practice relies on imperfect biochemical and imaging tools. The 2025 AASLD pediatric practice statement therefore recommends integrating hepatic assessment with cardiometabolic characterization and evaluating not only the presence of steatosis, but also disease severity, fibrosis risk, metabolic comorbidities, and the need for specialist management (7).

Alanine aminotransferase remains the most accessible screening test because it is inexpensive and easily repeated. However, ALT is not a direct measure of hepatic fat, inflammation, or fibrosis, and its interpretation is affected by biological variability, assay differences, intercurrent illness, and sex-specific thresholds (37). Clinically relevant steatosis may also occur despite ALT values within conventional laboratory reference ranges. Both the NASPGHAN guideline and the AASLD pediatric practice statement therefore position ALT as a useful component of assessment, but not as a sufficient stand-alone diagnostic or prognostic test (7, 36).

Conventional B-mode ultrasound is widely available and relatively inexpensive, but its diagnostic performance is limited by operator dependence, body habitus, machine settings, subjective interpretation, and reduced sensitivity for mild steatosis (38). It cannot reliably quantify liver fat or stage fibrosis and is therefore of limited value for longitudinal monitoring or treatment-response assessment (39). Ultrasound should consequently be interpreted alongside biochemical, anthropometric, and metabolic findings rather than used to exclude disease or determine severity in isolation (7).

MRI-proton density fat fraction provides accurate, reproducible, and quantitative assessment of hepatic fat and is particularly valuable in specialist care, research studies, and clinical trials (40). Pediatric studies have demonstrated reliability and diagnostic accuracy for grading steatosis (41, 42). Nevertheless, cost, scanner availability, protocol standardization, and access prevent its use as a universal screening modality.

Elastography-based techniques provide additional non-invasive information regarding steatosis and fibrosis risk. Transient elastography with controlled attenuation parameter can estimate hepatic fat and liver stiffness, but interpretation depends on fasting status, probe selection, body habitus, technical quality, and pediatric cut-offs (43). Pediatric studies support its potential role in risk assessment, although it should not be interpreted independently of the broader clinical and metabolic profile (40, 44). Vibration-controlled transient elastography and point shear wave elastography are also increasingly evaluated. Xu et al. (45) reported stronger diagnostic performance for VCTE than pSWE, particularly among children with obesity and larger waist circumference, but technical performance and thresholds may vary with age, adiposity, probe characteristics, and disease spectrum. Elastography should therefore support structured risk stratification rather than function as a single decisive test.

Magnetic resonance elastography assesses a larger liver volume and may be less affected by some limitations of ultrasound-based techniques. However, pediatric evidence remains less extensive than adult evidence, while cost, availability, technical expertise, and feasibility in younger children restrict implementation. MRE is currently best positioned as a specialist or research tool rather than a routine first-line investigation (46, 47).

Liver biopsy remains the reference standard for diagnosing steatohepatitis, staging fibrosis, characterizing pediatric histological patterns, and identifying alternative pathology (48). Its use should nevertheless remain selective because it is invasive, costly, affected by sampling variability and interobserver differences, and unsuitable for repeated monitoring. Biopsy is most appropriate when histological findings are expected to alter management, including suspected advanced disease, diagnostic uncertainty, atypical features, or concern for additional liver pathology (48, 49).

Serum fibrosis scores and emerging biomarkers remain incompletely validated in children. Adult-derived tools such as FIB-4 perform poorly in pediatric populations because age is embedded in the formula and because fibrosis patterns, aminotransferase distributions, platelet counts, and comorbidity profiles differ from those of adults (50). Pediatric-specific scores and biomarker panels may improve risk stratification, but few have demonstrated sufficient external validity, generalizability, and clinical utility for routine independent use (50). Machine-learning fibrosis models are similarly promising but require transparent development, external validation, calibration across populations, and evidence that their use improves clinical decisions and patient outcomes rather than only statistical performance (51). Other candidate biomarkers are being investigated to characterize the metabolic and inflammatory phenotype of pediatric MASLD. HOMA-IR is readily derived from fasting glucose and insulin and may provide clinically useful information on insulin resistance, although its interpretation is influenced by age, pubertal stage, and adiposity and it is not a liver-specific biomarker (52). Bile-acid profiling has also shown potential, with pediatric studies demonstrating alterations in primary and secondary bile acids and in their dynamic postprandial responses; selected bile-acid species have been associated with liver fat and may help discriminate children with NAFLD from metabolically healthy controls (53). Cathepsin D is another emerging marker of hepatic inflammation. In a study of 96 children with biopsy-proven NAFLD, plasma cathepsin D differentiated steatosis from hepatic inflammation with an AUROC of 0.94, increasing to 0.998 when combined with cytokeratin-18 (54). Despite these promising findings, bile acids, HOMA-IR, and cathepsin D should currently be regarded as adjunctive or investigational biomarkers rather than validated stand-alone tests for routine pediatric MASLD care.

A precision diagnostic pathway should therefore be multimodal and risk-adapted (Figure 3). In lower-risk children, repeated ALT measurement combined with anthropometric and cardiometabolic assessment may support initial evaluation and monitoring. Persistent biochemical abnormalities, severe obesity, type 2 diabetes, suspected fibrosis, diagnostic uncertainty, or discordant clinical features should prompt consideration of advanced imaging and specialist referral (7, 55). The aim is not to apply the most sophisticated test to every child, but to match diagnostic intensity to disease risk, clinical uncertainty, feasibility, and the likelihood that the result will change management.

**Figure 3:**
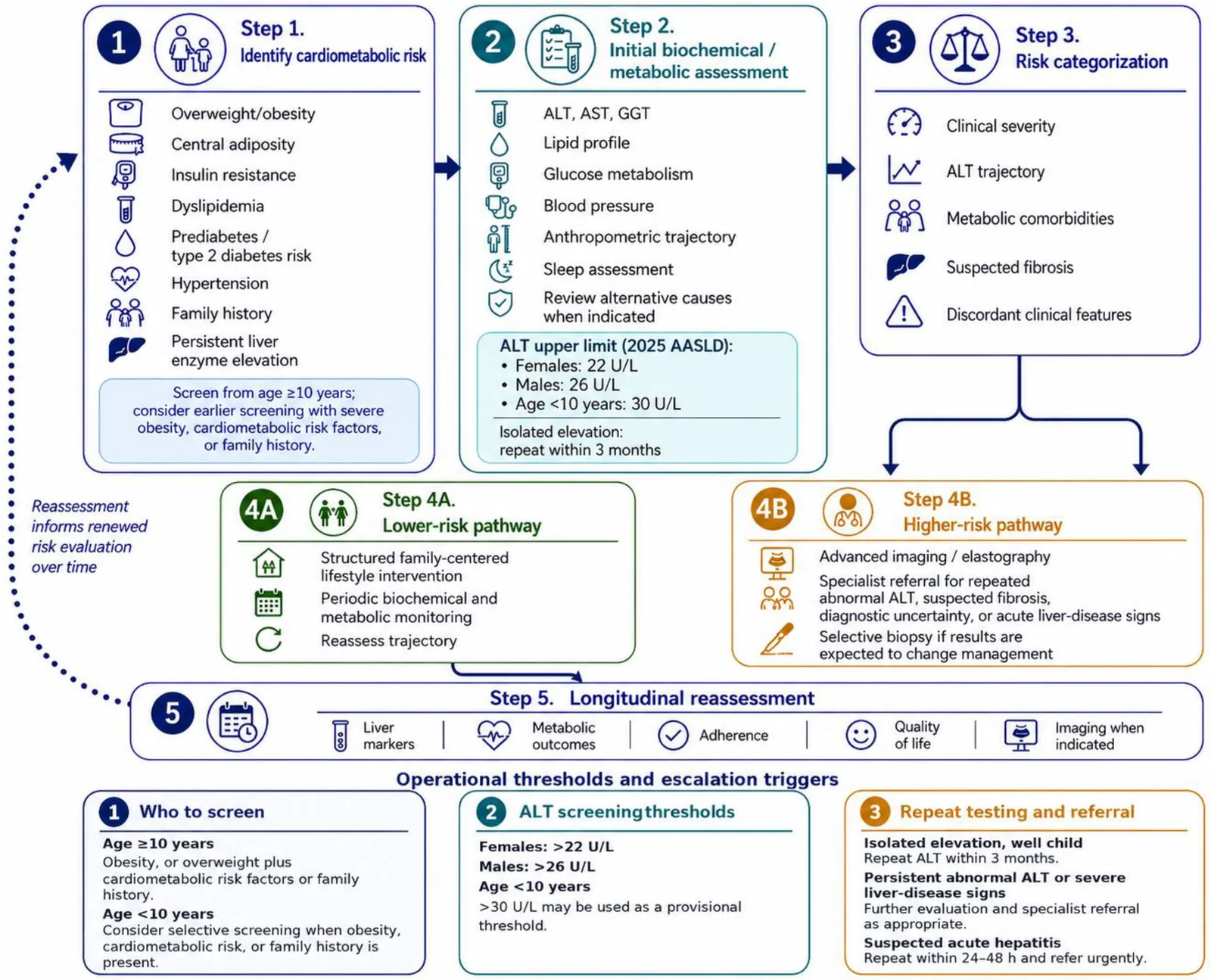
Proposed risk-adapted diagnostic pathway for pediatric MASLD. Conceptual, unvalidated pathway; prospective clinical validation is required. Thresholds adapted from the 2025 AASLD pediatric MASLD practice statement (7).

Future pediatric MASLD diagnosis may integrate imaging biomarkers, metabolic phenotyping, genetic susceptibility, gut–liver markers, digital-health data, and machine-learning prediction. These approaches should remain clinically subordinate to evidence of improved risk classification, management, feasibility, cost-effectiveness, and equitable access. The immediate priority is the development and external validation of pediatric-specific algorithms that combine accessible clinical information with targeted advanced testing for children at greatest risk (55).

The pathway shown in Figure 3 is a conceptual proposal and has not been prospectively validated. In children aged ≥10 years, ALT values >22 U/L in females and >26 U/L in males should be considered elevated; in selectively screened children aged <10 years, ALT >30 U/L may be used provisionally (7). An isolated elevation in an asymptomatic, otherwise well child should be repeated within three months. Persistent elevation should prompt evaluation for MASLD and alternative liver diseases, with specialist referral according to clinical context. Persistent ALT ≥2 times the sex-specific upper limit of normal, ≥44 U/L in females or ≥52 U/L in males, together with diagnostic uncertainty or findings suggestive of MASH or fibrosis should prompt specialist assessment and consideration of liver biopsy (7). Suspected acute hepatitis, jaundice, coagulopathy, rapidly rising aminotransferases, or other signs of severe liver disease require repeat testing within 24–48 h and urgent pediatric hepatology referral. Lower-risk children with improving ALT and cardiometabolic markers may continue family-centered intervention and periodic monitoring.

## 6 Prevention strategies in pediatric MASLD

Prevention is central to pediatric MASLD because the disease develops during periods of biological plasticity, behavioral formation, and family-dependent decision-making. Unlike adult MASLD, pediatric prevention may intervene before prolonged exposure to insulin resistance, ectopic fat accumulation, dyslipidemia, and systemic inflammation has occurred (55). Childhood and adolescence therefore represent critical windows for improving diet quality, physical activity, sedentary behavior, sleep, weight trajectory, and metabolic risk (56). Prevention should not, however, be reduced to weight loss alone. A broader cardiometabolic approach is required that addresses hepatic steatosis, insulin resistance, dyslipidemia, inflammatory risk, and the social conditions that shape health-related behaviors (7).

Family-centered prevention is particularly important because children have limited control over food purchasing, meal preparation, transport, opportunities for physical activity, sleep routines, screen exposure, and attendance at health-care appointments (57). These factors are largely determined by caregivers and constrained by household resources and the surrounding environment. Interventions directed only at the child are therefore unlikely to produce durable effects. Prevention should involve parents or caregivers, support household-level behavioral change, reduce blame and stigma, and recognize that adherence often reflects family capacity and environmental opportunity rather than individual motivation alone (7, 57).

Nutritional prevention should prioritize dietary quality, metabolic benefit, and long-term feasibility rather than rigid caloric restriction (15). Diets high in sugar-sweetened beverages, free sugars, refined carbohydrates, ultra-processed foods, saturated fats, and energy-dense nutrient-poor products may promote hepatic *de novo* lipogenesis, excess adiposity, dyslipidemia, and insulin resistance (15). In contrast, dietary patterns emphasizing vegetables, fruits, legumes, whole grains, fiber, fish, unsaturated fats, and minimally processed foods may support cardiometabolic and hepatic health. Systematic reviews suggest potential benefit from Mediterranean-style dietary patterns, although available studies remain heterogeneous in design, duration, adherence assessment, and liver-specific endpoints (13, 58).

The Mediterranean diet may be particularly suitable for long-term family adoption because it represents a flexible dietary pattern rather than a highly restrictive intervention. Its emphasis on plant-based foods, olive oil, fish, legumes, whole grains, nuts, and limited intake of highly processed foods provides a plausible basis for improving lipid metabolism, insulin sensitivity, oxidative stress, inflammation, and gut–liver axis function (58). Jamil et al. (58) reported potential improvement in liver-related outcomes, while Bernardino et al. (59) similarly supported its relevance in pediatric MASLD. However, both reviews emphasized that the available human evidence remains limited and methodologically diverse.

Reduction of free-sugar intake is another practical target. Fructose and other free sugars, particularly in sugar-sweetened beverages and processed foods, may increase hepatic *de novo* lipogenesis, energy intake, dyslipidemia, and insulin resistance (60). In a randomized trial of adolescent boys with NAFLD, Schwimmer et al. (61) found that an eight-week diet low in free sugars significantly reduced hepatic steatosis compared with usual diet. Nevertheless, the findings were preliminary and require confirmation in longer studies with clinically meaningful outcomes. Free-sugar reduction should therefore be incorporated into broader family-based dietary change rather than presented as an isolated intervention.

Physical activity contributes to prevention by improving insulin sensitivity, cardiorespiratory fitness, skeletal-muscle glucose uptake, adiposity distribution, and overall cardiometabolic health, even when weight loss is modest (62). A systematic review by Smith et al. (63) concluded that exercise interventions may improve pediatric liver-related outcomes, but the evidence was limited by small samples, heterogeneous protocols, inconsistent adherence, and variable outcome measures. Exercise should therefore remain a core preventive strategy, although no definitive pediatric MASLD prescription has yet been established regarding intensity, duration, frequency, supervision, or the optimal balance of aerobic and resistance activity.

Sedentary behavior should be addressed separately from structured exercise. A child may participate in planned physical activity while still accumulating prolonged sitting time, recreational screen exposure, irregular eating patterns, and poor sleep (64). Practical strategies include interrupting prolonged sitting, promoting active transport, incorporating movement into school and household routines, reducing energy-dense snacking during screen use, and encouraging family-based recreational activity (64). These approaches are consistent with an integrated model of behavioral and environmental modification rather than reliance on isolated exercise advice (57).

Sleep and circadian regularity should also form part of preventive assessment because short sleep duration, irregular timing, late-night eating, and obstructive sleep apnea may worsen appetite regulation, insulin sensitivity, obesity risk, inflammation, and adherence to other interventions (65). Sleep assessment is particularly relevant in children with severe obesity, snoring, daytime somnolence, or behavioral difficulties. Sleep disruption is unlikely to explain MASLD independently, but it may amplify the metabolic and behavioral conditions in which hepatic steatosis develops (65).

School and community environments are equally important because individual counseling has limited effect when children are exposed to food and activity environments that systematically promote metabolic risk. School meals, vending policies, health-literacy education, physical education, safe outdoor spaces, transport systems, food marketing, and neighborhood socioeconomic conditions influence the feasibility of prevention (66). Pediatric MASLD prevention should therefore be regarded as both a clinical and public health responsibility. Durable risk reduction requires family counseling alongside healthier food environments, reduced sugar-sweetened beverage exposure, improved opportunities for physical activity, and equitable access to preventive services (13).

Digital and mobile-health interventions may support self-monitoring, family engagement, nutrition education, physical activity, and continuity between clinical visits (67). However, their effectiveness should not be assumed solely because they are technologically innovative. Zhang et al. (68) described an mHealth-supported lifestyle intervention for children with overweight or obesity and NAFLD-related indicators, illustrating the potential of technology-assisted care. Such tools may be useful when they are developmentally appropriate, culturally sensitive, integrated with professional support, and designed to avoid widening inequalities among families with limited digital access or health literacy.

Precision prevention means adapting the timing, intensity, and content of intervention to the child's risk profile. Children with severe obesity, rapid weight gain, insulin resistance, dyslipidemia, early biochemical abnormalities, strong family history of type 2 diabetes, or suspected genetic susceptibility may require earlier and more intensive support (21). Nevertheless, precision prevention should not privilege advanced imaging or genetic testing over accessible preventive care. Its most immediate application lies in integrating anthropometry, metabolic markers, family history, lifestyle factors, and social determinants to identify children requiring greater intervention intensity and to address the barriers that determine whether prevention is feasible and sustainable (21).

## 7 Treatment of pediatric MASLD

Treatment of pediatric MASLD remains centered on structured lifestyle intervention, weight-management support when appropriate, and comprehensive cardiometabolic risk reduction. No MASLD-specific pharmacotherapy is universally approved for routine pediatric use. Therapeutic recommendations must therefore distinguish interventions that improve obesity or metabolic risk from those proven to modify pediatric liver-specific outcomes (14). Reductions in body mass index, alanine aminotransferase, or insulin resistance may be clinically meaningful but do not necessarily establish histological improvement, fibrosis regression, or long-term liver-related benefit (14). This distinction is especially important as incretin-based obesity treatments and adult MASH therapies continue to evolve (55).

Lifestyle intervention should be delivered as structured, multidisciplinary therapy rather than generic advice to “eat less and move more.” Management should integrate individualized nutritional counseling, physical activity, reduced sedentary behavior, sleep assessment, psychological support, caregiver involvement, and repeated follow-up. Therapeutic goals should extend beyond weight loss to include improvement in hepatic steatosis, aminotransferase trajectory, insulin sensitivity, lipid profile, blood pressure, physical fitness, quality of life, and long-term cardiometabolic risk (14). Pediatric NAFLD studies have demonstrated potential biochemical and histological improvement with lifestyle intervention, although response magnitude and durability vary considerably (8, 69).

Nutritional treatment should be adapted to the child's metabolic phenotype, cultural dietary pattern, family circumstances, food access, and practical barriers. Children with high sugar-sweetened beverage intake may require a different therapeutic emphasis from those affected primarily by irregular meals, large portion sizes, ultra-processed snacks, food insecurity, or limited household cooking capacity (70). Evidence supports improving overall dietary quality, reducing free sugars, and considering Mediterranean-style dietary patterns, but no single diet can currently be regarded as universally superior for all children with MASLD. Sustainable family-level dietary restructuring should therefore be prioritized over short-term restrictive prescriptions (69).

Physical activity should be prescribed with the same importance as nutritional therapy. Regular moderate-to-vigorous activity, reduced sedentary time and improvement in cardiorespiratory and muscular fitness should be adapted to the child's age, preferences, comorbidities, and family resources (71). Available exercise studies are promising but heterogeneous, preventing firm conclusions regarding the optimal modality, intensity, frequency, duration, or level of supervision (71). Recommendations should therefore be individualized and may include organized sport, walking, active transport, resistance-based play, school-based activity, or family movement routines.

Behavioral and psychological support is also essential because pediatric MASLD may coexist with obesity-related stigma, low self-esteem, emotional eating, anxiety, depression, family stress, body-image concerns, and reduced quality of life (4). Adolescents may experience additional adherence difficulties related to peer norms, school pressures, and increasing behavioral autonomy. Multidisciplinary treatment should therefore incorporate goal setting, motivational interviewing, problem solving, relapse planning, caregiver coaching, and stigma-sensitive communication rather than focusing exclusively on liver enzymes or weight (4).

Vitamin E and metformin remain among the most extensively studied pharmacological interventions in pediatric fatty liver disease. In the TONIC randomized controlled trial, neither intervention was superior to placebo for the primary endpoint of sustained ALT reduction (72). Vitamin E was nevertheless associated with improvement in selected histological features among children with biopsy-confirmed NASH. These mixed findings do not support universal use but may justify consideration of vitamin E in carefully selected specialist-managed cases after assessment of histological phenotype, safety, and family preference (72). Long-term high-dose vitamin E safety remains uncertain. Metformin should not be prescribed as a liver-directed therapy because it did not improve the primary TONIC outcome. It may still be indicated for impaired glucose metabolism, type 2 diabetes, or another endocrine condition, but this is distinct from MASLD-specific treatment (72). Medication adherence in adolescents is influenced by physiological, psychosocial, and clinical factors and should be incorporated into treatment planning (73).

Other antioxidant or hepatoprotective therapies have generated inconsistent evidence. The CyNCh trial found that delayed-release cysteamine bitartrate did not produce significant overall histological improvement compared with placebo in children with moderate-to-severe NAFLD (74). Similarly, the network meta-analysis by Yaser et al., which included 26 randomized trials and 1,503 pediatric participants, identified potential differences among interventions but did not establish a definitive standard pharmacological treatment (14). Vitamin D ranked highest for improvement in NAFLD Activity Score, with an estimated 1.62-point reduction, while vitamin E was the only intervention significantly associated with NASH resolution compared with placebo (RR 2.14, 95% CI 1.19–3.84). Delayed-release cysteamine ranked highest for ALT and AST reduction, although its estimated effect on fibrosis was not statistically significant. These comparative rankings should remain hypothesis-generating because the included trials varied in diagnostic criteria, interventions, doses, follow-up, and analytical methods. Certainty was not formally assessed using GRADE or CINeMA, only six trials used intention-to-treat analysis, four had high risk of bias, and ten raised some risk-of-bias concerns (14).

Anti-obesity pharmacotherapy has become increasingly relevant because obesity is a major disease driver and because adolescent obesity treatment has changed substantially. Liraglutide 3.0 mg daily and semaglutide 2.4 mg weekly are approved for chronic weight management in eligible adolescents aged ≥12 years, but neither is approved specifically for pediatric MASLD or MASH (75, 76). In the trial by Kelly et al., liraglutide produced an estimated between-group difference in BMI standard-deviation score of −0.22 at week 56. BMI reductions of at least 5% and 10% occurred in 43.3% and 26.1% of liraglutide-treated participants, compared with 18.7% and 8.1% receiving placebo (76). In STEP TEENS, mean BMI changed by −16.1% with semaglutide and +0.6% with placebo at week 68, while body-weight reduction of at least 5% occurred in 73% and 18% of participants, respectively (75). These findings demonstrate substantial obesity-directed efficacy but do not establish pediatric MASLD-specific histological improvement or fibrosis regression.

Safety, durability, feasibility, and access must be considered alongside efficacy. Gastrointestinal adverse events occurred in 64.8% of participants receiving liraglutide compared with 36.5% receiving placebo and in 62% receiving semaglutide compared with 42% receiving placebo (75, 76). In STEP TEENS, cholelithiasis occurred in 4% of semaglutide-treated participants and no placebo-treated participants, while discontinuation because of adverse events occurred in 5% and 4%, respectively (75). No clinically relevant differences in growth or pubertal development were identified during the 68-week trial, but this duration is insufficient to establish long-term safety throughout adolescence (75). The study also included only a seven-week off-treatment assessment and did not determine long-term durability after withdrawal.

The adult STEP 1 extension showed that participants previously receiving semaglutide regained 11.6 percentage points of body weight during the year after withdrawal, equivalent to approximately two-thirds of their prior weight loss, compared with 1.9 percentage points in the placebo group (77). These adult findings cannot be quantitatively extrapolated to adolescents but emphasize the need to discuss treatment duration, discontinuation, and possible weight regain before initiating therapy. Real-world adherence may also be affected by injection burden, gastrointestinal tolerability, family support, medication availability, insurance or reimbursement restrictions, and out-of-pocket costs (78–80). Such barriers may widen socioeconomic inequalities in access to effective obesity care (79–81). Treatment plans should therefore define the indication, intended outcomes, monitoring schedule, criteria for continuation or discontinuation, and management of interruption or weight recurrence (78, 79).

The evolving adult MASH therapeutic landscape may inform future pediatric drug development but should not be directly extrapolated to children. Resmetirom received accelerated FDA approval in 2024 for adults with noncirrhotic MASH and moderate-to-advanced fibrosis, while semaglutide 2.4 mg received accelerated approval in 2025 for adults with MASH and moderate-to-advanced fibrosis (82–85). Neither is approved specifically for pediatric MASLD or MASH. An ongoing randomized phase 2 trial is evaluating semaglutide 2.4 mg in young people with obesity, dysglycemia, and MASLD and includes intrahepatic triglyceride content as a liver-related endpoint (NCT05067621) (86). Pediatric adoption will require direct evidence regarding liver-specific efficacy, pharmacokinetics, growth, pubertal development, treatment duration, and long-term safety.

Metabolic and bariatric surgery may be considered for highly selected adolescents with severe obesity and clinically significant comorbidities, but it should not be presented as routine MASLD treatment (78). Xanthakos et al. (87) reported a high prevalence of NAFLD among adolescents undergoing bariatric surgery, demonstrating the substantial liver-disease burden associated with severe obesity. Surgery may improve weight, insulin resistance, and obesity-related comorbidities, but requires strict eligibility criteria, multidisciplinary assessment, psychological evaluation, long-term nutritional monitoring, and careful transition planning (88, 89). Its role lies within comprehensive severe-obesity care rather than as an isolated liver-directed intervention.

Treatment response should be monitored using multiple domains because no single marker captures the full clinical effect. ALT trends are useful but may not accurately reflect resolution of steatosis, histological improvement, or fibrosis regression (7, 55). Monitoring should also include weight trajectory, waist circumference or other adiposity measures where available, glucose metabolism, lipid profile, blood pressure, sleep-related symptoms, physical fitness, quality of life, and, in selected cases, imaging-based assessment of hepatic fat or stiffness (7, 55).

A precision-treatment model should align intervention intensity with disease severity, metabolic phenotype, comorbidities, developmental needs, family capacity, and social context. Children with mild steatosis, stable liver enzymes, and improving metabolic markers may receive structured family-based support and periodic monitoring. Those with severe obesity, type 2 diabetes, persistent ALT elevation, suspected fibrosis, or inflammatory-fibrotic features may require specialist hepatology and endocrinology assessment and more intensive multidisciplinary treatment (21). The metabotypes described by Huneault et al. (17) provide a conceptual basis for risk-adapted care, although prospective validation and pragmatic clinical algorithms are required. Precision treatment is therefore not limited to medication selection; it involves matching the type and intensity of care to each child's biological, metabolic, developmental, familial, and social risk profile.

Because pediatric MASLD requires coordinated management of liver disease, obesity, metabolic dysfunction, behavioral factors, and family-level barriers, Figure 4 summarizes a multidisciplinary care model centered on the child or adolescent and family.

**Figure 4:**
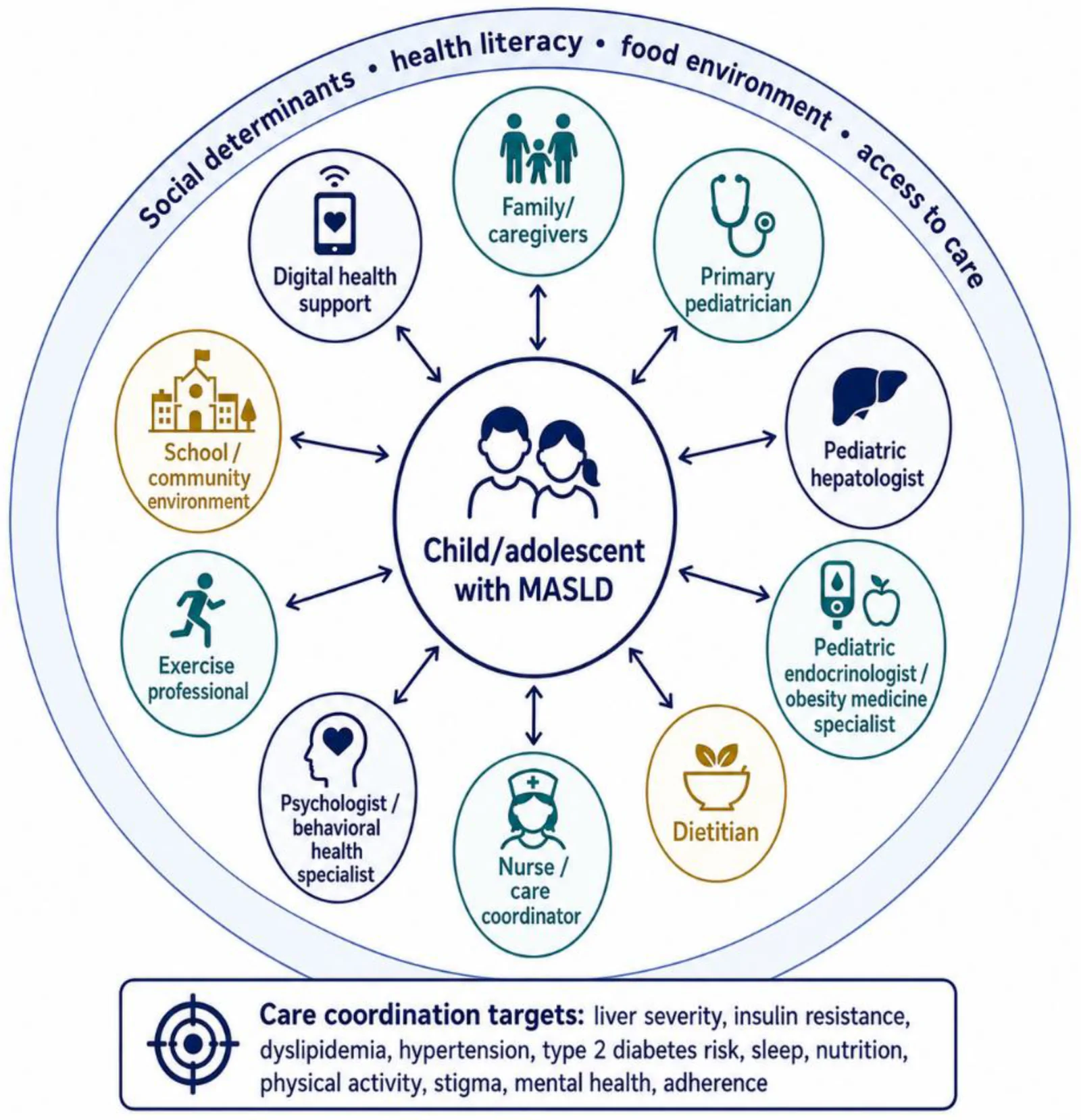
Multidisciplinary care model for pediatric MASLD.

Pediatric MASLD management should follow a multidisciplinary, family-centered model that places the child or adolescent and caregivers at the center of care. The team may include the primary pediatrician, pediatric hepatologist, endocrinologist or obesity-medicine specialist, dietitian, nurse or care coordinator, psychologist, exercise professional, and, where appropriate, school, community, and digital-health support. This approach recognizes pediatric MASLD as a systemic cardiometabolic condition shaped by family routines, lifestyle behaviors, psychosocial factors, and structural determinants of health. Care coordination should address liver disease severity, insulin resistance, dyslipidemia, hypertension, type 2 diabetes risk, sleep, nutrition, physical activity, stigma, mental health, and long-term adherence.

## 8 Conclusion

Pediatric MASLD is a heterogeneous, developmentally embedded, cardiometabolic liver disease that requires more than simple detection of hepatic steatosis. The transition from NAFLD to MASLD provides an opportunity to reframe the disease around metabolic dysfunction, reduce exclusionary terminology, and integrate liver assessment with broader cardiometabolic care. Recent evidence demonstrates a substantial burden among children with overweight and obesity, while emerging data on metabotypes, genetics, microbiome signatures, imaging biomarkers, and machine-learning approaches suggest that pediatric MASLD is not a uniform condition. These developments support a shift toward risk-adapted and phenotype-informed care, while also highlighting the need for validation, caution, and equity in implementation.

At present, lifestyle intervention, family-centered care, nutritional improvement, physical activity, behavioral support, and multidisciplinary cardiometabolic risk management remain the foundation of treatment (90). Pharmacological options, including obesity-directed therapies, are promising in selected adolescents but should not yet be presented as established MASLD-specific therapies without pediatric liver-specific evidence. The future of pediatric MASLD care will depend on validated non-invasive diagnostics, longitudinal cohorts, rigorous intervention trials, precision phenotyping, ethical use of digital and omics tools, and health-system models that address both biological and social determinants of disease. A precision medicine approach should therefore aim not to complicate care unnecessarily, but to deliver the right intensity of prevention, diagnosis, and treatment to the right child at the right time.
